# Efficacy of Nitric Oxide Fumigation for Controlling Codling Moth in Apples

**DOI:** 10.3390/insects7040071

**Published:** 2016-12-02

**Authors:** Yong-Biao Liu, Xiangbing Yang, Gregory Simmons

**Affiliations:** 1 USDA-ARS, Crop Improvement and Protection Unit, Salinas, CA 93905, USA; 2 Department of Vegetable Crops and Weed Science, University of California, Salinas, CA 93905, USA; xbya@ucdavis.edu; 3 USDA-APHIS-PPQ-CPHST, Salinas, CA 93905, USA; Gregory.S.Simmons@aphis.usda.gov

**Keywords:** postharvest pest control, quarantine treatment, postharvest quality, *Cydia pomonella*

## Abstract

Nitric oxide (NO) fumigation under ultralow oxygen (ULO) conditions was studied for its efficacy in controlling codling moth and effects on postharvest quality of apples. NO fumigation was effective against eggs and larvae of different sizes on artificial diet in 48 h treatments. Small larvae were more susceptible to nitric oxide than other stages at 0.5% NO concentration. There were no significant differences among life stages at 1.0% to 2.0% NO concentrations. In 24 h treatments of eggs, 3.0% NO fumigation at 2 °C achieved 100% egg mortality. Two 24 h fumigation treatments of infested apples containing medium and large larvae with 3.0% and 5.0% NO resulted in 98% and 100% mortalities respectively. Sound apples were also fumigated with 5.0% NO for 24 h at 2 °C to determine effects on apple quality. The fumigation treatment was terminated by flushing with nitrogen and had no negative impact on postharvest quality of apples as measured by firmness and color at 2 and 4 weeks after fumigation. This study demonstrated that NO fumigation was effective against codling moth and safe to apple quality, and therefore has potential to become a practical alternative to methyl bromide fumigation for control of codling moth in apples.

## 1. Introduction

Codling moth, *Cydia pomonella* (L.) (Lepidoptera: Tortricidae), is a major pest of pome fruits, stone fruits, and walnuts in the USA. Furthermore, it is a quarantine pest in some other countries and, therefore, a major trade barrier to the export of infested products such as apples to international markets including Japan and the Republic of Korea. Exported apples from codling moth occurring regions to Japan are currently required to be treated to control codling moth. A combination treatment of a 55-day cold storage followed by a 2-h fumigation with 56 g/m^3^ of methyl bromide at 10 °C is needed to achieve complete control of codling moth in apples [1]. Methyl bromide fumigation treatment that controls codling moth eggs can cause unacceptable phytotoxic effects on apples [2]. Phosphine fumigation is not very effective against codling moth and is projected to take 5 day to achieve 99% larval mortality at 0.5 °C [3]. Safe and effective alternative treatments are needed to control codling moth in apples and other agricultural products. A recently discovered new fumigant, nitric oxide, may have the potential to control codling moth in apples.

Nitric oxide is a ubiquitous cell signal molecule in most organisms and modulates a wide range of biological and physiological processes [4,5]. In insects, nitric oxide plays roles in diverse physiological processes including reproduction, locomotion, olfaction, learning and memory, and host defense mechanisms [6,7]. Nitric oxide is produced naturally by almost all types of organisms including bacteria, fungi, plants, and animals, as well as by lightning and fossil fuel combustion such as automobiles and power plants. Nitric oxide is also produced on a massive scale as an intermediate in the syntheses of nitric acid from ammonia.

Recently, nitric oxide was discovered to be a potent fumigant against insects [8]. Nitric oxide is highly effective against all insects at various life stages including egg and pupa. Treatment time ranges from a few hours to three days depending on insect species, life stages, nitric oxide concentration, treatment time, and temperature. Efficacy of nitric oxide fumigation increases with increased concentration, treatment time, and temperature. Eggs and pupae are more tolerant to nitric oxide fumigation than mobile life stages [8].

Because nitric oxide reacts with oxygen spontaneously to form nitrogen dioxide, nitric oxide fumigation must be conducted under ultralow oxygen (ULO) conditions to minimize its oxidation. For fresh products, nitric oxide fumigation in most cases will also need to be terminated by flushing with nitrogen to dilute nitric oxide prior to exposing products to ambient air to minimize exposure of the products to nitrogen dioxide. When terminated properly with nitrogen flushing, nitric oxide fumigation is safe to postharvest quality of all tested fresh products. In addition, nitric oxide fumigation for pest control was also found to enhance postharvest quality of strawberries [9,10]. Nitric oxide fumigation also does not leave harmful residues [11]. In this paper, codling moth on artificial diet and in apples was studied for responses to nitric oxide fumigation and an effective nitric oxide fumigation treatment was also tested on apples to determine its impact on apple quality.

## 2. Materials and Methods

### 2.1. Insect

A codling moth colony, originated from a laboratory mass-rearing culture of the Okanagan-Kootenay Sterile Insect Release Program (Osoyoos, BC, Canada), was maintained on modified Steward pink bollworm calco red diet [12] in plastic containers at 24–28 °C, 16:8 (L:D) photoperiod, and 50%–80% RH at USDA-APHIS (Animal and Plant Health Inspection Service)-Plant Protection and Quarantine (PPQ)-Center for Plant Health Science and Technology (CPHST) laboratory in Salinas, CA, USA. Moths were collected in 24 h after enclosure and set up in 20–30 pairs in a mesh wire cage for mating and oviposition. Moths were supplied with 7.5% sucrose solution in a saturated cotton wick as food and a sheet of wax paper as oviposition substrate (egg sheet). The egg sheet with ≤24 h old eggs was collected and used for experiments. Larvae of different sizes (small, medium, and large) based on length were also collected from the rearing media for experiments.

### 2.2. Fumigation Procedures

All nitric oxide fumigation treatments of codling moth were conducted under ULO conditions of ≤30 ppm O_2_ in 1.9 L glass jars. Oxygen levels were monitored using an oxygen analyzer (Series 800, Illinois Instruments, Inc., Johnsburg, IL, USA). The jars loaded with insects were flushed with nitrogen to reduce oxygen to or below the desired level as described before [8]. Nitric oxide gas at 99.5% purity in a pressurized cylinder from an industry vendor was released in a foil bag and was then injected into the jars using an airtight syringe. The syringe and its connected tubing were flushed with nitrogen before being used to inject nitric oxide. Nitric oxide concentrations were based on volumes of nitric oxide gas injected over the volume of the jars. The jars were then held at 2 °C in an environmental chamber for the duration of the treatment to complete the fumigation and were then vented in a fume hood to terminate the treatment.

### 2.3. Fumigation of Eggs and Larvae on Artificial Diet

Two sets of fumigation tests were conducted. In the first set of tests, eggs and larvae of different sizes were fumigated together for 48 h to determine their relative susceptibilities to nitric oxide. In the second set of tests, only eggs were fumigated for 24 h to determine an effective treatment. In the first set of tests, egg sheets of <24 h were collected and cut into pieces with about 30–50 eggs on each piece. They were then placed individually in small plastic vials (3 cm in diameter by 7 cm in height). A piece of yellow sticky tape was suspended in each vial to trap neonates hatched from eggs. The vials were sealed with screened lids. Larvae of different sizes: small, medium, and large were collected from rearing media which were set up at different times and were set up separately by size in a group of 10 in small screened cages with small amount of diet. Eggs and larvae of all sizes were then placed in 1.9 L fumigation jars to be fumigated with nitric oxide.

Fumigations with 0.5%, 1.0%, and 2.0% NO of 48 h were conducted under ≤30 ppm O_2_ at 2 °C to determine effects on mortality of codling moth eggs and larvae. In each test, two vials (for eggs) and two cages (for each larval size) were fumigated for each treatment. Untreated eggs and larvae were also set up under ULO and normal atmosphere and held at 2 °C as controls during the experiment. Each treatment was replicated three times. After fumigation, larvae were incubated at 22 °C and 90%–95% RH overnight before being scored for mortality. Eggs were incubated under the same conditions as stated above for at least 2 weeks to allow all viable eggs to hatch and neonates trapped on the sticky cards. Neonates caught on the sticky cards and unhatched eggs were then counted to determine egg mortality. A total of 2034 eggs, 370 small larvae, 388 medium larvae, and 347 large larvae were tested.

In the second set of tests, codling moth eggs were fumigated with 0.5%, 1.0%, 1.5%, 2.0%, and 3.0% NO for 24 h under ≤30 ppm O_2_ at 2 °C to determine an effective 24 h treatment. Eggs were set up in small plastic vials as described above. In each test, 2–3 vials with eggs were fumigated in each treatment. Controls under ULO and air were also included in each test. Each treatment was replicated 3–6 times. A total of 3427 eggs were used. Eggs mortality was scored as described above.

### 2.4. Fumigation of Infested Apples

Medium sized codling moth larvae from the rearing medium were removed and placed on apples for infestation. Apple sizes were selected to be ≤7 cm in diameter to fit through the opening of the 1.9 L jars. Larvae were reared on apples for one week. After one-week infestation, larvae had medium to large sizes and were well established inside the apples. The infested apples were then placed in 1.9 L jars (1–2 apples/jar) and held at 2 °C in a refrigerator to be acclimatized overnight. An ULO atmosphere with ≤30 ppm O_2_ was then established in each jar with nitrogen flush and nitric oxide gas was then injected into the jar to start a fumigation treatment. Fumigation treatments of 24 h with 3.0% and 5.0% NO were conducted at 2 °C. Controls under both ULO and the normal air were included in each test. After fumigation, the apples were held at room temperature for one day before being dissected to collect larvae and determine their mortality. In each test, the two nitric oxide fumigation treatments were replicated twice. The test was replicated over 20 times. A total of 221 infested apples were used.

### 2.5. Fumigation of Sound Apples

Sound apples from supermarket were fumigated with 5.0% NO under ULO conditions for 24 h at 2 °C to determine potential effects on visual quality of apples for marketing. Sound apples (var. Granny Smith) were obtained from local supermarkets and inspected for any imperfections. Apples without any visual defects were selected for fumigation test. Colors of randomly selected apples were measured using a spectrometer (ColorTec-PSM, Accuracy Microsensors, Inc., Pittsford, NJ, USA). The color parameters including luminosity (L*), chroma (*C*), and hue angle (*H*°) were measured. Each apple was measured three times on surface. Apples were then cut into halves and firmness of the cut surface of each half was then measured three times using a penetrometer with a 3 mm round tip (QA Supplies, LLC., Norfolk, VA, USA). The firmness was measured as grams of force to penetrate the flesh. For both color parameters and firmness, average values for each apple were used in the statistical analyses. The rest of selected apples free from defects were then randomly separated into two groups, one for the fumigation treatment and the other used as controls.

In the fumigation treatment, apples were loaded into a 23 L chamber modified from a pressure cooker. About 2 kg of apples were fumigated in the chamber in each test. The chamber loaded with apples was flushed with nitrogen to establish an ULO atmosphere with ≤30 ppm O_2_. An aliquot of 1000 mL of nitric oxide gas from the foil bag was injected into the fumigation chamber in a fume hood. The chamber was then held at 2 °C in a walk-in cooler for 24 h to complete the fumigation treatment. The chamber was then flushed with nitrogen gas at 5 LPM flow rate for 20 min in the fume hood to dilute nitric oxide in the fumigation chamber. A flue gas monitor with a 5000 ppm NO sensor (Kane 900Plus, Kane International Ltd., Hertfordshire, UK) was used to monitor NO levels and the chamber was opened to terminate the fumigation treatment after the NO level dropped below 50 ppm. Apples from the control were stored at 2 °C during fumigation. After fumigation, apples from both the treatment and the control were stored in separate plastic boxes in a walk-in cooler and visual quality and firmness were evaluated at 2 and 4 weeks. Procedures for post-treatment quality evaluations were the same as described above. Apple colors and firmness for both the treatment and the control were measured. The fumigation test was replicated six times. A total of 25 kg of apples were used.

### 2.6. Data Analysis

Mortality data for eggs and larvae were transformed by arcsine√x prior to analysis of variance. For comparisons among egg and larvae of different sizes, mortality rates among the life stages at each NO concentration were subjected one-way ANOVA and Tukey HSD multiple range test. For the 24 h fumigations of eggs, mortalities for different NO concentrations were also subjected to one-way ANOVA and Tukey HSD multiple range test. Apple quality data including firmness and the color parameters at the start of fumigation and 2 and 4 weeks after fumigation were compared between the treatment and the control. Regression lines over time were constructed for the average values of firmness and the color parameters for the treatment and the control to determine their trends of variation over time. The slopes of the two regression lines for the treatment and the control for each parameter were also compared using a *t*-test to determine the differences between the treatment and the control in the trends of variation of the postharvest quality traits. All statistical analyses were carried out using the fit model platform of JMP Statistical Discovery software [13].

## 3. Results

Nitric oxide fumigation treatments were effective against both eggs and larvae of codling moth on artificial diet in 48 h treatments at 2 °C (Table 1). However, there were some variations among egg and larvae of different sizes. The small larvae were most susceptible to nitric oxide fumigation treatments as indicated by the significantly higher mortalities at 0.5% and 1.0% NO concentrations as compared with other life stages. There was no significant difference among eggs, medium sized larvae, and large sized larvae at any nitric oxide concentration. Complete control of both eggs and larvae was achieved with the 2.0% NO fumigation treatment (Table 1). In the 24 h fumigations of codling moth eggs, egg mortality increased significantly with increasing nitric oxide concentrations and complete control of eggs was achieved in the fumigations with 2.0% and 3.0% NO (Table 2). Egg mortalities in the controls under the normal atmosphere and ULO were about 25% and were not significantly different. Nitric oxide fumigation was effective against codling moth larvae in infested apples. The 5.0% NO fumigation treatment of 24 h achieved complete control of codling moth larvae and 3.0% NO fumigation achieved 98% larval mortality. Controls had low mortalities of 3.5% to 4.8% (Table 3).

**Figure 1:**
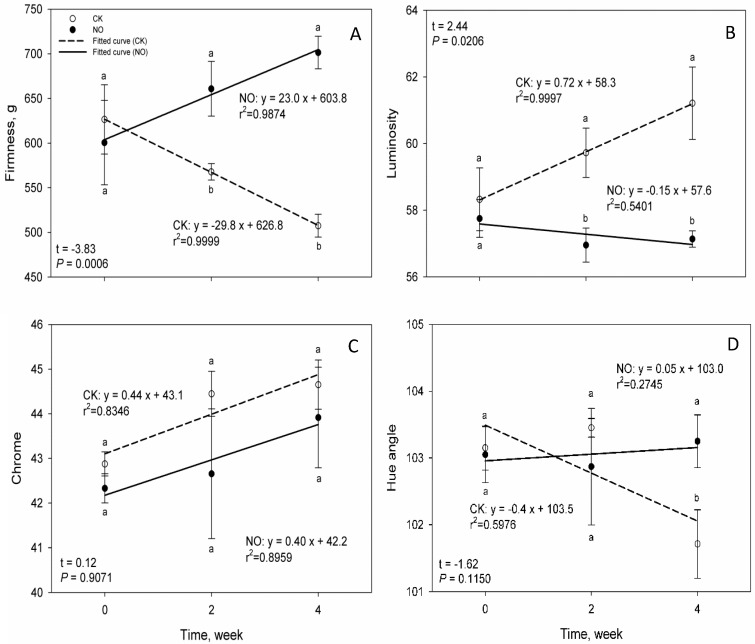
Correlations of firmness, luminosity, chroma, and hue angle of apples from the nitric oxide fumigation (NO) and the control (CK) with post-treatment storage time at 2 °C and comparisons between the fumigation treatment and the control. The four graphs represented the four parameters and were denoted as **A**, **B**, **C**, and **D** respectively. For each parameter, the two values for the treatment and the control at a same time followed by different letters were significantly different at *P* ≤ 0.05 based on *t*-test [13]. The statistical values of *t* and *P* for each parameter were for comparison of slopes of the regression lines for the treatment and the control.

Nitric oxide fumigation for control of codling moth was not only safe to apple quality but also enhanced some postharvest quality traits (Figure 1). At the start of fumigation, there were no significant differences in firmness or color parameters between the treatment and the control. There were also no injuries or stains on apples immediately after fumigation. At 2 and 4 weeks after fumigation, apples from the treatment had significantly higher firmness as compared with the control. Over the course of 4 week post-treatment cold storage, the firmness of apple flesh from the treatment actually showed a trend of increase over time while the firmness for the controls declined. The two regression lines diverged and their slopes differed significantly (Figure 1A). The luminosity of apples from the treatment showed slight decline over time as indicated by the small negative slope of the regression line. The luminosity for the control, however, showed considerable increases over time. There was also a significant difference between the two regression lines in slope. The luminosity values for the control were significantly higher than those for the treatment at 2 and 4 weeks post-treatment (Figure 1B). Chroma values for both the treatment and the control were not significantly different at any time and showed a trend of increases over time (Figure 1C). The hue angle for the treatment remained stable over time. However, hue angle for the control declined considerably over time even though the slopes of the two regression lines were not significantly different. At 4 weeks, the hue angle for the control became significantly lower as compared with that for the treatment (Figure 1D).

## 4. Discussion

Nitric oxide fumigation was effective against both eggs and larvae of codling moth and complete control of eggs and larvae of different sizes were achieved. The effectiveness of nitric oxide fumigation against codling moth added new data on efficacy of NO fumigation against insects and demonstrated that nitric oxide is effective against both eggs and mobile life stages. This differs from other fumigants such as sulfuryl fluoride and phosphine. Sulfuryl fluoride is not very effective against insect eggs especially at low temperatures [14]. Phosphine is also not effective to some pests due to either resistance or tolerance. Phosphine fumigation can also take many days to achieve effective control of some pests [15,16]. Therefore, neither of the two fumigants is a good choice for postharvest pest control. In comparison, nitric oxide fumigation is not only effective against all life stages but also has relatively shorter treatment times as compared with phosphine.

The 24 h treatment time to control codling moth larvae in infested apples demonstrated that nitric oxide can penetrate deep into fruits and is effective to control internal pests. However, it is not clear if nitric oxide can effectively penetrate other fruits such as peaches, pears, and oranges to control internal pests in them, and research is needed to determine whether nitric oxide fumigation treatment needs to be developed for each fruit species or variety.

Nitric oxide fumigation has been reported to enhance postharvest quality of fresh fruit and vegetables due to its antagonistic effects on ethylene biosynthesis [15]. In the current study, fumigated apples were significantly firmer than the controls and this indicates that nitric oxide fumigation helps to maintain the firmness of apples. Color parameter luminosity (L^*^), chroma (*C*), and hue angle (*H°*) represent lightness, brightness, and color tone respectively. The significant increases in luminosity for the control indicated that apples from the control were lighter than the fumigated apples. Hue angles of 90° and 180° represent yellow and green. The significant reduction in hue angle at 4 weeks from about 103° for the control indicated that color tone of the control apples varied slightly toward yellowish over time. This was likely due to losses of chlorophyll and also a cause for the higher luminosity of the control apples.

Nitric oxide is a messenger chemical with multitude functions in organisms. In fruits, nitric oxide functions as an inhibitor of ethylene biosynthesis and delays fruit ripening [17]. The enhancement effect of nitric oxide fumigation on apple quality is likely caused by its inhibitory effects on biosynthesis of ethylene, which promotes fruit maturity and senescence. The reduced firmness and increases in lightness as well as reduced hue angle in the control apples were consistent with effects of ethylene. Therefore, it was likely that nitric oxide fumigation for codling moth control effectively prevented ethylene induced maturation process and helped to preserve apple quality. These results were consistent with studies from other laboratories [18,19] as well as our study on nitric oxide fumigation of strawberries in which fumigated strawberries had higher firmness and bright and richer color as compared with controls [10]. In addition, NO has been reported to inhibit surface browning of cut apples [20,21,22].

The economics of nitric oxide fumigation has been discussed earlier [8,9]. Even with the added costs of nitrogen, nitric oxide fumigation has a moderate cost and will likely be cost effective in postharvest pest control as compared with most methods used currently. As nitrogen generating equipment is readily available and nitric oxide gas is also available from multiple vendors, nitric oxide fumigation is also expected to be technically feasible to be developed and used commercially. Even though nitric oxide is a natural chemical from most organisms and has been used to alleviate certain medical conditions, it will still need to be registered as a chemical pesticide in order to be used in the United States since its mode of action for pest control is unknown and it is documented to be toxic to humans. Once it becomes registered, nitric oxide has potential to become a preferred and practical alternative fumigant for postharvest pest control on fresh as well as stored products given its many advantages as discussed above. More research efforts are needed to develop effective nitric oxide fumigation treatments on wide varieties of insects and mites on different commodities and to develop large treatment protocols for commercial applications to hasten the practical applications of nitric oxide fumigation.

## 5. Conclusions

Nitric oxide fumigation was found to be effective against codling moth eggs and larvae at low temperature. Complete control of eggs and larvae on artificial diet was achieved in 24 h and 48 h treatments. Complete control of larvae in infested apples was also achieved in 24 h fumigation with 5.0% nitric oxide. The 5.0% NO fumigation for codling moth control had no negative impact on apple quality. Therefore, nitric oxide fumigation has potential to be an alternative treatment for codling moth in apples.

## Figures and Tables

**Table 1:** Susceptibility of codling moth eggs and larvae of different sizes on artificial diet to 48 h nitric oxide fumigation treatments at 2 °C.

NO (%)	Life Stage	Total Insects	Mortality (%) (Mean ± SE)	ANOVA
0.5	Egg	313	60.5 ± 6.3ab	df = 3, 36
	Larva—small	62	90.6 ± 5.1a	*F* = 4.38
	Larva—medium	65	44.1 ± 13.9b	*P* = 0.01
	Larva—large	59	63.5 ± 10.1ab	
1.0	Egg	348	92.1 ± 4.8a	df = 3, 36
	Larva—small	63	100a	*F* = 1.17
	Larva—medium	64	89.1 ± 6.0a	*P* = 0.34
	Larva—large	58	93.0 ± 2.7a	
1.5	Egg	346	97.7 ± 1.6a	df = 3, 36
	Larva—small	64	100a	*F* = 0.99
	Larva—medium	64	100a	*P* = 0.41
	Larva—large	58	100a	
2.0	Egg	342	100	
	Larva—small	64	100	
	Larva—medium	64	100	
	Larva—large	58	100	
C (ULO)	Egg	346	32.8 ± 4.5a	df = 3, 36
	Larva—small	57	12.7 ± 7.8b	*F* = 6.89
	Larva—medium	65	6.3 ± 4.7b	*P* = 0.001
	Larva—large	57	12.5 ± 9.4b	
Control	Egg	339	30.9 ± 5.4	df = 3, 36
	Larva—small	60	14.6 ± 9.3	*F* = 2.67
	Larva—medium	66	18.8 ± 11.1	*P* = 0.06
	Larva—large	57	14.1 ± 9.3	

Mortality data were transformed by arcsine√x prior to analysis of variance. For each treatment, mortality rates followed by different letters were significantly different based on Tukey HSD multiple range test (*P* ≤ 0.05) [13].

**Table 2:** Responses of codling moth eggs to 24 h nitric oxide fumigation treatments at different concentrations at 2 °C.

NO (%)	Total Eggs	Mortality (%) (Mean ± SE)	ANOVA
0.5	585	39.6 ± 3.9c	df = 6, 69
1.0	580	44.1 ± 4.2bc	*F* = 135.66
1.5	148	68.3 ± 4.2b	*P* < 0.0001
2.0	586	100a	
3.0	443	100a	
Control (ULO)	529	24.7 ± 4.2d	
Control	556	25.2 ± 4.5d	

Egg mortality data were transformed by arcsine√x prior analysis of variance. The values followed by different letters were significantly different based on Tukey HSD multiple range test using the Fit model of the JMP statistical discovery software at *P* ≤ 0.05 [13].

**Table 3:** Effects of 24 h nitric oxide fumigation treatments at 2 °C on mortality of codling moth larvae in apples.

NO (%)	Apples	Total Larvae	Mortality (%) (Mean ± SE)	ANOVA
3.0	77	388	98.0 ± 0.9a	df = 3, 214
5.0	76	386	100a	*F* = 1719.07
C (ULO)	37	162	4.8 ± 1.6b	*P* < 0.0001
Control	31	128	3.5 ± 1.7b	

Mortality data were transformed by arcsine√x prior to analysis of variance. For each treatment, mortality rates followed by different letters were significantly different based on Tukey HSD multiple range test (*P* ≤ 0.05) [13].
